# Assessment of the Corrosion Behavior of Friction-Stir-Welded Dissimilar Aluminum Alloys

**DOI:** 10.3390/ma15010260

**Published:** 2021-12-30

**Authors:** Rami Alfattani, Mohammed Yunus, Ahmed F. Mohamed, Turki Alamro, Mohamed K. Hassan

**Affiliations:** 1 Department of Mechanical Engineering, Umm Al-Qura University, Makkah City 24372, Saudi Arabia; rafattni@uqu.edu.sa (R.A.); afmohamed@uqu.edu.sa (A.F.M.); tsamro@uqu.edu.sa (T.A.); mkibrahiem@uqu.edu.sa (M.K.H.); 2 Mechanical Engineering Department, Faculty of Engineering, Sohag University, Sohag 82524, Egypt; 3 Production Engineering & Design Department, Faculty of Engineering, Minia University, Minia 61111, Egypt

**Keywords:** friction stir welding, dissimilar alloys, corrosion resistance, weight loss, submersion and salt spraying tests

## Abstract

The fuel consumption of high-density automobiles has increased in recent years. Aluminum (Al) alloy is a suitable material for weight reduction in vehicles with high ductility and low weight. To address environmental problems in aircraft and maritime applications, in particular rust development and corrosion, the current study assesses the corrosion behavior during friction stir welding (FSW) of two dissimilar Al alloys (AA6061 and AA8011) in various corrosive conditions using salt spraying and submersion tests. Two acidic solutions and one alkaline solution are used in these tests, which are performed at room temperature. The two specimens (AA6061 and AA8011) and the weld region are suspended in a salt spraying chamber and a 5 wt.% NaCl solution is continually sprayed using the circulation pump for 60 h, with the specimens being weighed every 15 h to determine the corrosion rates. According to the salt spraying data, the weld zone has a higher corrosion resistance than the core components. For twenty-eight days, individual specimens are submerged in 3.5 wt.% HCl + H_2_O and H_2_SO_4_ + H_2_O solutions and seawater. The weld area specimens exhibit stronger corrosion resistance than the base material specimens, and weight loss in the saltwater medium is lower when compared to the other test solutions, according to the corrosion analysis. The scanning electron microscope (SEM) analysis demonstrates that the base metal AA8011 is considerably corroded on its surface.

## 1. Introduction

In comparison to high-strength steel, mild steel is inferior. Al alloys have melting temperatures of 480 °C to 660 °C and have higher thermal stability than steel. A modest number of alloys are added to pure Al during the casting process (sand, die, investment casting, etc.) to improve the strength and casting properties. Sand casting is used to make most Al alloys, including Al–silicon alloys, Al–magnesium alloys, Al–zinc alloys, and Al–copper alloys. Aluminum alloys are very effective materials worldwide because they have high strength-to-weight ratios, great thermal and electric conductivity, and outstanding corrosion resistance (CR) in marine environments as compared to other materials. Recycling aluminum alloys is easy. They are lightweight material with high weldability, machinability, formability, and ductility and with non-magnetic, and non-toxic characteristics. Al alloys have a lower modulus than steel, require unique welding processes due to their abrasive nature, and are also costlier than steel. A thin Al oxide layer on the metal’s surface protects it against oxidation even when it is exposed to air. Aluminum alloys are utilized in a variety of applications, including aircrafts, automobiles, marine applications, electrical conductors, overhead transmission lines, construction, and for household items including cans, foils, cooking utensils, and window frames. Pressure and non-pressure welding, depending on the technique to be used in the weld joints (e.g., heat or pressure), are used to connect similar or dissimilar materials. Because the base metals do not melt, pressure welding (e.g., diffusion and friction stir welding) involves applying external pressure to two pieces to force them to coalesce at temperatures below melting, without the need for a filler material and without changing the original material characteristics. In a previous study, dissimilar Al alloy welding was performed using the FSW technique with several eccentric tools, which yielded a maximum tensile strength (5% increase) using a 0.8 mm pin due to the enhanced grain edges. The maximum eccentric tool pin delivers high hardness by improving the grain structure of the weld nugget zone and enlarging the boundary [1].

On the advancing weld side, the tensile testing of dissimilar weld joint specimens (6061-T6 and 7075-T6) demonstrated fracture in the heat-affected zone (HAZ) of the AA6061 material. The tool offset is the most important factor in generating heat in the tool pin, and it controls the thermophysical properties of the weld region [2]. The FSW procedure achieved high hardness in the weld zone by using a two-sided, flat, cylindrical pin tool and a tapered cylindrical pin. The tapered pin-produced components had a greater CR throughout all welding zones, with the thermomechanically affected zone (TMAZ) having the lowest CR according to the microstructure analysis.

Various challenges and concerns that have arisen as a result of the FSW procedure. Friction stir welding has the advantage of being able to weld harder alloys with high wear resistance and reduced tool costs. On the advancing side of the material, a narrower nugget zone was seen in a previous study, reducing joint strength [3]. The FSW of AA2014-AA6061 dissimilar Al alloy joints led to a lower temperature on the AA6061 weld side and a poorer weld zone structure. The strength of the tensile specimen rose with increasing welding speed (90 to 300 mm/min), resulting in varied nugget forms, whilst high temperatures resulted in onion ring profile nuggets [4]. When compared to similar Al alloys, the FSW of dissimilar materials (AA5052-AA6061) showed that their mechanical characteristics were enhanced. In another study, the microstructural examination showed that the intermixing of base materials in the nugget zone is not homogenous, resulting in decreased hardness [5]. Because of the deep pin penetration in the welding, the tool pin height was increased. Joint faults are caused by improper mixing of base materials when less heat is generated in the weld by utilizing a lower shoulder-to-pin diameter (D/d) ratio for the tool [6]. With a maximum Vickers hardness of 1220 rpm tool rotation and a welding rate of 40 mm/min, the FSW marine-grade Al alloy produced a superior weld connection. The joint strength is influenced by the fine grain structure achieved in the center of the weld region as a result of dynamic recrystallization [7].

Further, it is well established that the advancing side material is generally found in the nugget zone or weld portion’s middle region, where the hardness curves of both base materials have the same pattern but the weld hardness curves somewhat differ [8,9]. When the square pin profile of the tool was utilized compared to other profiles of the pin in the welding of AA2024 (advancing side) and AA5083 [10], the welding speed was shown to be a crucial element in enhancing the tensile strength of the joints at all rotational speeds (1120–1400 rpm). The tensile strength of the material failure was discovered in the HAZ of the AA2219 (retreating side) with low hardness in dissimilar weld joints (AA2219-AA5083), and an elevated CR was seen in comparison to comparable weld joints. The greater hardness levels were found in the material’s stir zone [11]. The threaded tool pin, maximum welding speed, tool tilt angle, and minimum tool rotational speed are the most significant factors determining the tensile strength [12]. By involving taper hexagonal and taper cylindrical pins in multi-output optimization, both tensile and hardness values were maximized. The welding speed was the most important element in this study, followed by the rotational speed and tool profile [13]. At optimum conditions (1200 rpm tool rotating speed and 80 mm/min welding rate), maximum tensile strength, hardness, and elongation of 12.5% were achieved [14]. Even under varied heat-generating conditions, the dissimilar FSW joints with different tool profiles (cylindrical, triangular, and square) generated the highest tensile strength and hardness values [15]. When submerged in 3.5 percent NaCl solution, the CR of the weld zones of base materials (AA5083, AA7023) had a low potential voltage and strong polarization resistance. Because of the difference in volta potential measurements, the current density of the base material AA7023 was larger, and the boundary between the two base materials was severely attacked [16].

The corrosion potential of Al alloy 6061 was smaller than that of HT590 in polarization CR testing of dissimilar materials. The cathode shield protected the HT590 and Al alloy 6061 in the polarization test due to the formation of an oxide film on the materials [17]. The Al alloy (6061) samples manufactured using FSW and gas tungsten arc welding (GTAW) were examined after submersion for 30 min in a 3.5% NaCl solution. The FSW samples had a higher CR than the GTAW samples, although the weld zones of both welded samples showed lower CR values. Because the fine grains developed the fine limit in the stir zone of the FSW process, the CR of the weld area decreased [18]. The CR and weight loss of the parent metal (AA7022) revealed that it had an outstanding CR for pitting corrosion, while in the welding region the parent metal had a smaller number of shallow corrosion pits than the parent metal [19]. The pitting corrosion of AA1100 and AA5083 materials, when exposed to seawater in a weight loss technique, revealed that AA1100 had the best CR, making it acceptable for marine applications [20]. In a submersion test with a 1% acetic acid solution, the Al had a higher CR than the AA5754. The use of laurel oil lowers the current density while increasing the polarization resistance. The Al alloy had a less cracked structure than the Al alloy [21]. The CR the of materials in TMAZ was outstanding in diverse corrosive media, such as 3.5 wt.% NaCl, 3.5 wt.% NaCl + 1 wt. percent HCl, and exfoliation corrosion solution utilizing submersion and electrochemical methods at the stir zone. The lowest mechanical characteristics and higher mass loss were observed in samples in EXCO solution [22]. In 3.5 wt.% NaCl solution, the CR levels of the Al alloys prepared using FSW, shielded metal arc welding (SMAW), and GTAW were very low and were moderate in SMAW samples. FSW samples had the lowest corrosion rate (Cr) and the highest CR. Increasing the welding speed (from 63 to 100 mm/min) in a 3.5 percent NaCl solution enhanced the CR and lowered the Cr of the weld zone and the HAZ when compared to the base materials [23].

Through submersion tests in seawater with and without an inhibitor (sodium benzoates), it was determined that the CR of a marine application material (AA2024) prevented weight loss. The inhibitor in seawater promoted a higher Cr to Al alloy ratio and a higher CR [24]. Under galvanic corrosion tests, the CR values of the FSW AZ31B Mg alloy and Al6063 alloy in a 3.5% NaCl solution varied over time, while the weight loss of the weld zone sample was greatest, followed by the AZ31B Mg alloy and Al6063 material [25]. The FS-welded AA2024 was evaluated in a salt spraying chamber with NaCl solutions with various pH values, with the pH value of 7 providing maximum CR for the materials, which showed pit shapes in SEM images with increasing spraying duration [26]. Electrochemical and submersion techniques were used to determine the CR values of different Al alloy joints. In the weld region, the pitting corrosion was uniform. The weld nugget zone of a dissimilar joint showed higher deterioration during the galvanic corrosion test. Because of microstructural modification after welding, the HAZ suffered the most corrosion. For the AA6056 material, the microstructural changes accelerated the CR [27]. The dynamic recrystallization generated by the increased welding speed lowered the grain size of the weld zone and modified the grain boundary angle, indicating that the HAZ grains were due to the coarse appearance. The mechanical characteristics of dissimilar FSW joints were produced using different tool speeds while keeping all other parameters constant. At low tool rotation speeds, the SEM examination revealed large numbers of pits and voids in the fractured areas of the samples (lower surface of the weld region). The joint strength grew as the speed increased, although it failed at the HAZ on the AA6061 side [28]. Few cavity defects were noted in the stir zones of dissimilar weld joints (AA6061 and AZ31 Mg) in the SEM images because of the insufficient frictional heat of the Mg alloy and metal oxides caused by the thermal interaction with the base material and oxygen, which accelerated the Cr [29].

The majority of researchers have used comparable FSW procedures, particularly with Al alloys from the 5xxx, 6xxx, and 7xxx series, but did not use distinct FSW processes with Al alloys from the most recent series (AA8xxx). The literature search also showed that the FSW of dissimilar alloys, such as AA6061 and AA8011, has not been investigated in terms of corrosion behavior, prompting the current study. The welding of dissimilar materials using traditional welding methods results in poor weldability and material alterations in terms of the chemical, mechanical, and thermal characteristics. As it prevents electromagnetic radiation and UV production during welding, FSW is considered an environmentally beneficial approach for overcoming these challenges. Based on several studies on CR in FSW, this study will evaluate the corrosion characteristics of FSW Al alloys (AA6061 and AA8011) using a salt spraying test and a laboratory submersion test. The weld region’s CR is measured and the microstructural characteristics are investigated using OM and SEM. Furthermore, this research focuses on CR analysis to assess the influence of delamination, pores, grooves, and debris during and after the FSW process, utilizing the microstructural characteristics of corrosion test samples to reveal their impacts.

## 2. Methods and Materials

Friction stir welding is a robust welding procedure that is widely employed in the automobile, aeronautical, shipbuilding, and food industries, involving both similar and dissimilar welding processes. In this present research work, the corrosion features of the FSW of dissimilar Al alloys are evaluated using a novel methodology. Because the 6xxx and 8xxx series Al alloy materials have higher corrosion resistance than other Al alloy series, the current research is mostly focused on the use of AA6061 and AA8011 to create a compact structure with better strength. Both dissimilar materials were commercially procured in the form of plates utilized to prepare specimens for experimental purposes. They are both appropriate for use in lightweight structures and are easy to form into varied shapes due to their ductile nature. A precision saw cutting machine with efficient coolant was used to cut all of the specimens from the plates, with final dimensions of 100 × 50 × 4 mm^3^. Table 1 shows the chemical patterns for these alloys with enhanced mechanical properties (MP) developed from the two base materials, AA6061 and AA8011, and from the cataloged Al alloys series used for this study.

### 2.1. Friction Stir Welding (FSW) Process

**Figure 1:**
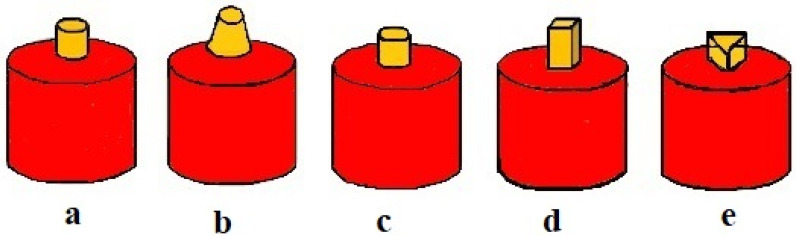
Different profiles of cylindrical FSW tools with (**a**) straight, (**b**) tapered, (**c**) threaded, (**d**) square, and (**e**) triangular pins.

The tool’s shoulder and pin play important roles in the FSW process, as they are the key factors involved in producing effective welding joints. Figure 1 [30] shows the various shapes of FSW tool pins that were used to successfully manufacture weld connections. The shoulder contact area provides the consistent force, the plunging pin blends the edges of the two materials and homogeneous material, while the tool stirring process provides strength. For the present corrosion study, a straight cylindrical pin tool was utilized to weld the specimens with standard dimensions (20 mm shoulder diameter, 5 mm cylindrical diameter, and 3.7 mm pin length). High-carbon, high-chromium steel was selected for the tool because of its high wear and abrasion resistance.

Table 2 lists the welding process parameters for this experiment, which were selected based on a review of various research articles, as well as the machine conditions and materials that were welded [31].

**Figure 2:**
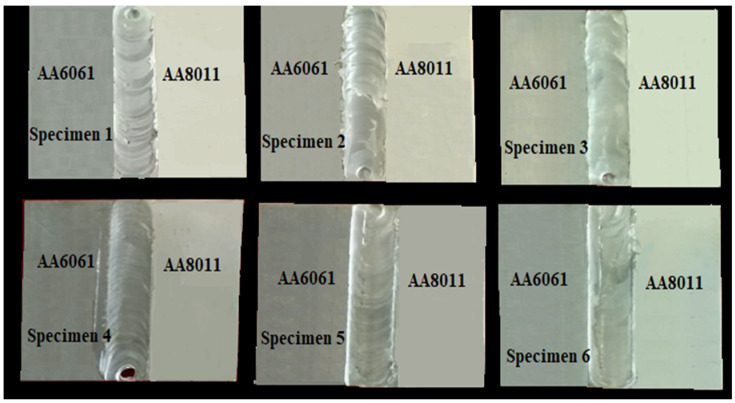
Dissimilar specimens after welding.

The FSW process is the result of a combination of mixing, stirring, and applying force repeatedly in the transverse direction, both on the advancing and retraction sides, with the help of a spinning tool. A vertical CNC machine with a cylindrical tool pin was used to weld dissimilar specimens in this application. Fixture clamps with Allen screws were used to secure specimens of dissimilar alloys on the worktable. Both specimens were clamped in the same direction, and the revolving tool pin rotated and stirred the materials, ensuring that the base material was uniformly intermixed. Figure 2 demonstrates how the tool rotational speed and transverse speed allowed straight and continuous welding of the specimen, and how the welding was performed securely and accurately.

**Figure 3:**
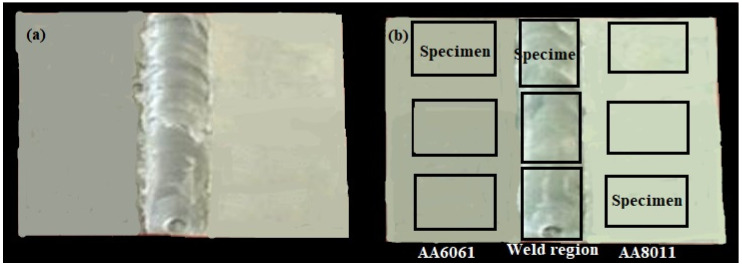
(**a**) Friction-stir-welded specimen and (**b**) corrosion specimens marked within a welded specimen.

Figure 3a shows a specimen welded using the FSW method. Three specimens were selected from AA6061, the weld region, and AA8011 for slicing. The specimens (18, 12, and 4 mm, respectively) were measured using the salt spraying and submersion tests and are depicted in Figure 3b.

**Figure 4:**
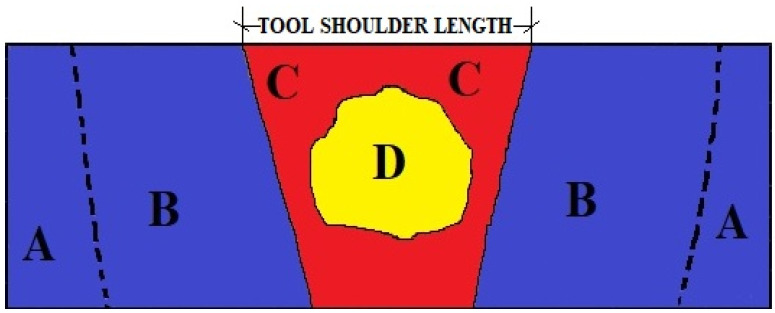
Microstructural zones.

In the FSW procedure, the microstructure zones of the specimens are subdivided as depicted in Figure 4: (A) the unaffected zone (BUZ) of the base material is positioned far from the WNZ, where it is unaffected by heat or deformation and maintains all of its properties; (B) the heat-influenced zone (HAZ) is closer to the heat-affected WNZ, but there is no plastic deformation owing to changes in microstructure and mechanical properties (similar to the fusion welding process); (C) the thermomechanically impacted zone (TMAZ) is influenced by heat and material deformation caused by friction and mechanical movement of the tool, affecting the microstructural and mechanical characteristics; (D) the weld nugget zone (WNZ) is located right beneath the tool shoulder contact zone, where the FSW tool generates a lot of heat, causing considerable deformation and resulting in fine grains replacing the original grains.

### 2.2. Corrosion Analysis Tests

Al has a high CR compared to other materials due to the existence of a protective oxide layer. The primary objective of this study was to assess the corrosion rates (Crs) of AA6061 and AA8011, as well as the weld area, in hostile media. Two procedures were chosen to estimate the Cr—a salt spraying test and a laboratory submersion test. The corrosion test was designed to determine the material’s corrosion resistance in a variety of settings, including basic, saltwater, and acidic conditions. This corrosion test is mostly used for the failure analysis of industrial and construction materials. There are several forms of corrosion, including atmospheric, uniform, pitting, erosion, stress, galvanic, fretting, fatigue, and intergranular corrosion. The CR is immediately determined in salt spraying and laboratory submersion tests by reducing the error. These tests are simple to execute for quick screening of specimens. Therefore, the salt spraying test and laboratory submersion test were chosen in the present work.

#### 2.2.1. Salt Spray Corrosion Test

The salt spraying corrosion test is one of the traditional methods used to analyze the CR of materials. Specimens measuring 18 × 12 × 4 mm^3^ were prepared and cleaned thoroughly before being loaded into the salt spraying chamber. The salt spraying test parameters are presented in Table 3. The specimens of each material (AA6061, A8011, and weld area) were suspended in the salt spraying chamber for 60 h following ASTM B 117-16.

Using a circulation pump, the specimens were constantly sprayed with a 5 wt.% sodium chloride solution for 60 h. To measure the corrosion impacts produced by salt spraying, the weight of the specimen was tested every 15 h. At the end of the 60 h experiment, the specimens were taken from the salt spraying chamber, rinsed in clean distilled water to remove salt deposits from the surface, and dried immediately. The weight of the test specimen was determined using a computerized electronic scale. The difference between the initial weight of each specimen before salt spraying and the final weight of the same specimen after salt spraying was used to compute the weight loss.

#### 2.2.2. Laboratory Submersion Test

The laboratory submersion corrosion test was deemed to be more reliable and generate fairly accurate results when compared to prior corrosion tests. This form of testing makes it easy to spot and reject materials that are not suitable for a certain application. This is the most efficient and cost-effective method of evaluating material choices. The results of the other tests were available in a short time but were less precise.

The ASTM G3 standard was followed for the submersion test. For the submersion test, three test mediums were used. Two of the media were acidic, namely 3.5 wt percent HCl + H_2_O and 3.5 wt percent H_2_SO_4_ + H_2_O, and one was alkaline seawater. The test specimens measured 18 × 12 × 4 mm^3^ and were constructed from the base materials (AA6061 and AA8011) and from the weld area. The pH values of the two acidic solutions and the seawater used in the submersion test were determined using a digital pH meter.

The weight percentages of various corrosion test solutions, the submersion time temperatures, and the pH values of the solutions are shown in Table 4. In the submersion test, the weight loss technique was employed. The specimens were polished and cleaned with acetone before being immersed in distilled water and air-dried. The starting weight of each specimen was computed before the submersion test. The specimens from the AA6011 and AA8011 base materials and from the weld area were submerged in HCl solution, H_2_SO_4_ solution, and seawater in glass beakers. The specimens were kept at 32 °C for 28 days. At the end of the submersion test, the specimens were taken from the immersion solution and dried completely (after 28 days). The final weights of the test specimens were obtained using a digital scale.

**Figure 5:**
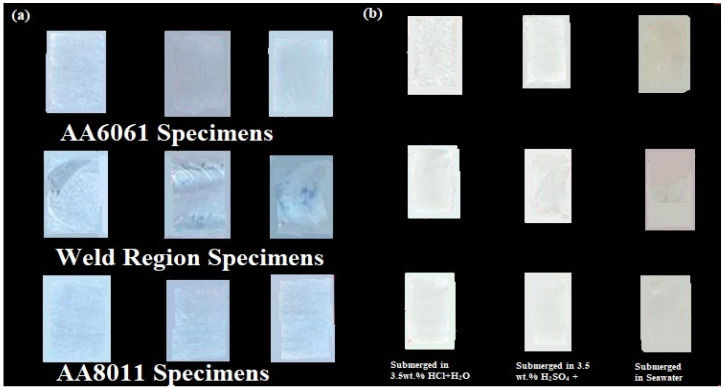
Corrosion test specimens: (**a**) before corrosion test; (**b**) after corrosion test.

The difference between the initial weight of each specimen before the submersion test and the final weight of the same specimen after the submersion test was used to compute the weight loss of each specimen after 28 days. The surfaces of the basic materials AA6061 and AA801, as well as that of the weld zone, are shown in Figure 5a, b before and after corrosion. The corroded portions of the specimens can be seen in Figure 5b. The basic materials were significantly affected in all corrosive conditions, as can be seen in the figure. On the other hand, the weld area surface was slightly eroded, suggesting that the weld region specimen had high corrosion resistance. All of the submerged surfaces were altered to a whitish color, except in the seawater medium. The seawater medium formed an ash-colored corrosion layer on the submerged surfaces of the specimens, which provided good corrosion resistance. The weight loss was calculated using both the salt spraying and submersion experiments. Equation (1) was used to compute the corrosion rate (mm/yr) based on the weight loss.

Cr = 87.6 × (ΔW/ρat) mm/yr(1)

where C_r_ is the corrosion rate in mm/yr (weight loss measurements), K = 87.6, ΔW is the weight loss in g (W_b_ − W_a_), ρ represents the density values of the base materials (2.7 and 2.71 g/cm³), a is the area of the specimen in cm^2^, and t represents the specimen submersion time in hours.

## 3. Results and Discussion

In this section we discuss the corrosion and its impact on the Al alloy base materials and weld zone. The materials were subjected to a salt spraying test and a basic laboratory submersion test. The variance of corrosion in the examined samples was assessed using SEM pictures of corroded surfaces.

### 3.1. Salt Spraying Test Results

The weight loss was greatest in the base material of AA8011 (0.24 g), moderate in AA6061 (0.148 g), and minimal in the weld area specimen according to the results of the salt spraying test (0.12 g). Similarly, the Cr of the welded region was determined to be the lowest (0.00264 mm/yr), whereas the highest Cr was for base material AA8011 at 0.00516 mm/yr and the corrosion rate for base material AA6061 was 0.00324 mm/yr (Table 5).

**Figure 6:**
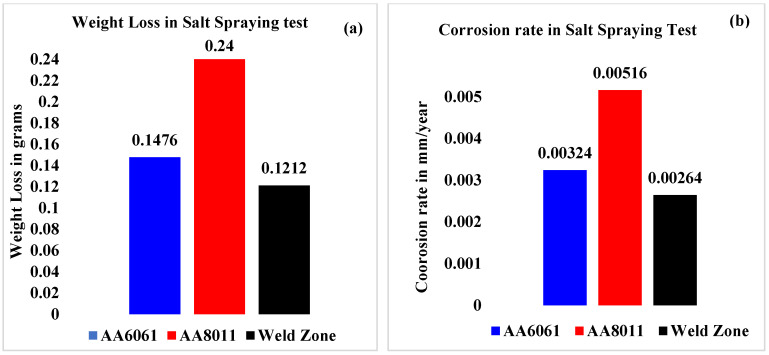
(**a**) Weight loss values and (**b**) corrosion rates of the base materials and weld region in salt spraying test.

The weight loss sustained by the three specimens after completing the salt spraying test for 60 h is depicted in Figure 6a. The weld zone had a higher CR (low weight loss) than the base materials. Figure 6b shows the corrosion rates of the three portions of the FSW joint after 60 h of salt spraying. The Cr of the weld zone was lower than the rates of the base materials, with the highest value being found for AA8081.

### 3.2. Submersion Test Results

The results of the laboratory submersion test are reported in terms of the samples weight before and after submersion, weight loss caused by the corrosion solutions, and the Cr values for each specimen (Table 6).

**Figure 7:**
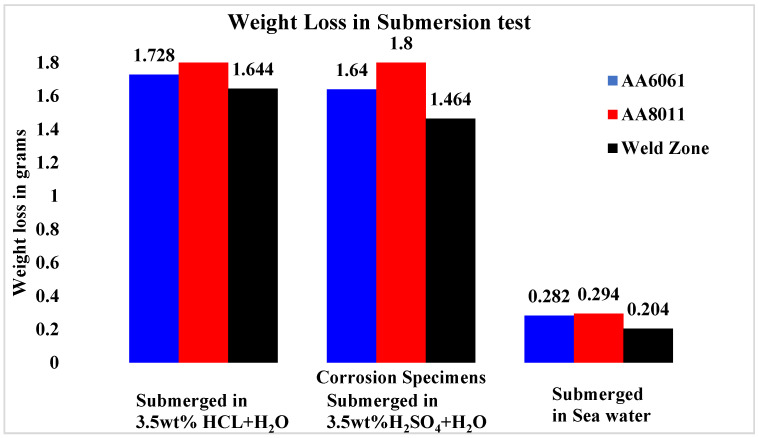
Weight loss values for base material and weld region specimens after 28 days of submersion in three different solutions.

The results of the submersion test demonstrated that acid media such as HCl and H_2_SO_4_ are more corrosive than alkaline media such saltwater, as shown in Figure 7. Among the acid media studied here, H_2_SO_4_ was more corrosive than HCl. In every test medium, the weld area specimen presented the greatest CR values. Weight loss in the weld zone was minimal for all three specimens studied, ranging from 1.644 g (HCl) to 1.464 g (H_2_SO_4_) in acid conditions and up to 0.204 g in alkaline media (seawater). For the base materials, weight loss was greater in acid media, ranging from 1.728 g (HCl) to 1.64 g (H_2_SO_4_), while in alkaline media (seawater) the values ranged from 0.282 g (AA6061) to 0.294 g (AA8011).

**Figure 8:**
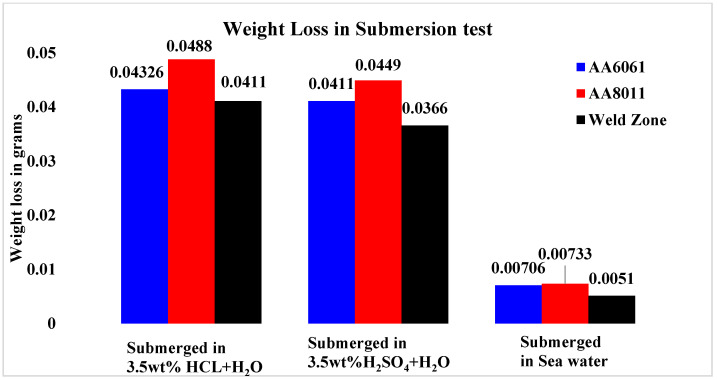
The corrosion rates of the base material and weld region specimens after 28 days submersion in three different solutions.

Figure 8 shows the Cr values for the base material and weld zone specimens after 28 days of immersion in three different solutions. The Cr values for the base materials in acid media varied from 0.0448 mm/yr (HCl) to 0.0449 mm/yr (H_2_SO_4_), whereas the Cr values for the base materials in saltwater ranged from 0.00706 mm/yr (AA6061) to 0.00733 mm/yr (AA6061) (AA8011). The grain size of the base materials was homogeneous; nevertheless, the grain sizes varied throughout the rolling process, resulting in dislocation defects. Recrystallization creates homogenous equiaxed grains when base materials are regularly intermixed during welding. The recrystallization of grains leads to improved mechanical and thermal characteristics, as well as fewer faults in the weld zone, which had a higher CR. In similar alloys, recrystallization occurs in the weld region, although this does not provide cathodic protection, which is why less corrosion resistance is seen in the weld regions in dissimilar alloys. Moreover, seawater can also cause higher Cr values, making base metals prone to higher metal loss.

### 3.3. Weight Loss in Submersion Test

**Figure 9:**
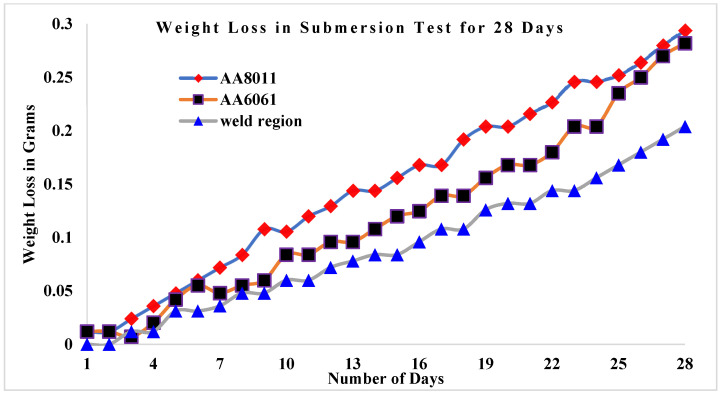
Weight loss rates for base materials and weld region sample against the number of days submerged in seawater.

**Figure 10:**
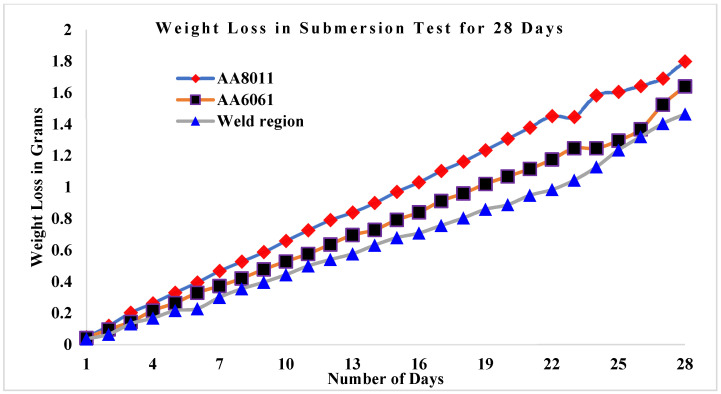
Weight loss rates for base materials and weld region sample against the number of days submerged in 3.5 wt.% HCl + H_2_O.

**Figure 11:**
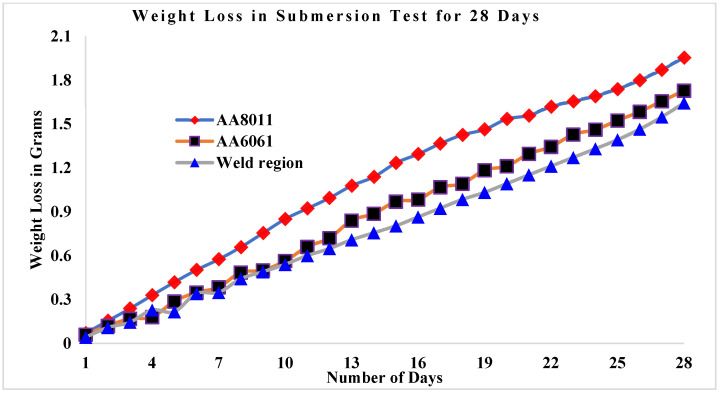
Weight loss rates for base materials and weld region sample against the number of days submerged in 3.5 wt.% H_2_SO_4_ + H_2_O.

Figure 9 shows the weight loss values for the three corrosion specimens as a function of the number of days they were submerged in saltwater. After being immersed for 28 days, base material AA6061 was found to gradually lose weight. The weld area specimen lost the least weight in saltwater, changing from 11.42 to 11.216 g, as indicated by weight monitoring at one-day intervals over the 28 day submersion period. The AA8011 base material lost the most weight, changing from 11.51 to 11.216 g, while the AA6061 base material changed from 11.5 to 11.218 g. Figure 10 showed a link between weight loss and submersion days in HCl solution in three cases. At the 28 day interval, the weight loss of the AA8011 specimen submerged in HCl medium was estimated to be between 11.51 and 9.71 g, which was higher than predicted. The weight loss in the weld region was estimated to be between 11.42 and 9.956 g. AA6061 began at 11.5 g and concluded at 9.87 g. The weight loss rates for the three test specimens submerged in H_2_SO_4_ solution for 28 days are displayed in Figure 11. The AA8011 material dropped weight progressively from 11.51 to 9.554 g, the AA6061 material lost weight gradually from 11.5 to 9.772 g, and the weld area sample lost weight gradually from 11.42 to 9.7764 g. In all submersion media, the weight loss rates for the base materials (AA6061 and AA8011) were found to be greater than the weight loss of the weld region specimen.

The weight loss rates were greater in the base materials (AA6061 and AA8011) than in the weld area specimen in all submersion media, demonstrating that denser grains induce higher CR [32].

### 3.4. Microstructure Analysis by SEM

**Figure 12:**
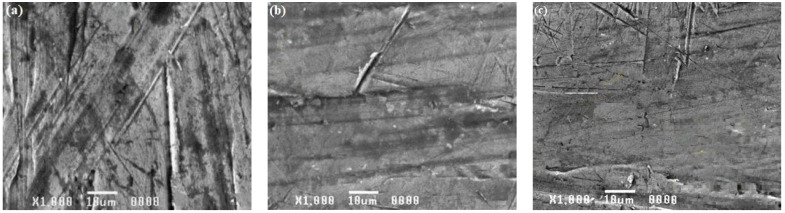
SEM images of (**a**) AA6061, (**b**) AA8011, and (**c**) weld region specimens after submersion testing in seawater.

**Figure 13:**
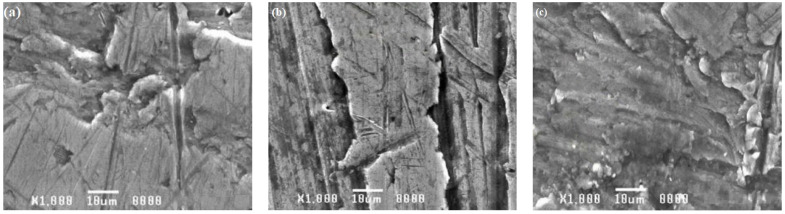
SEM images of (**a**) AA6061, (**b**) AA8011, (**c**) and weld region specimens after submersion testing in H_2_SO_4_.

**Figure 14:**
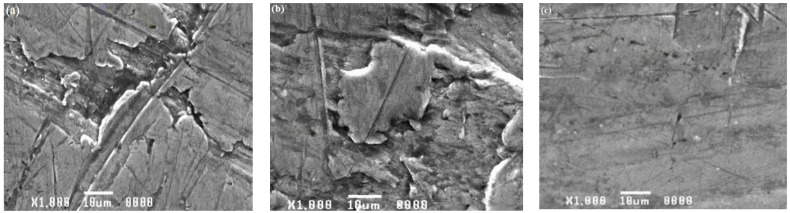
SEM images of (**a**) AA6061, (**b**) AA8011, (**c**) and weld region specimens after submersion testing in HCl.

For the base material and weld area specimens exposed to corrosion tests in both acid and alkaline solutions, a scanning electron microscopic investigation was conducted. Figure 12, Figure 13 and Figure 14 demonstrate how the influence of corrosion on the surfaces of the tested specimens was analyzed and evaluated using SEM images.

SEM images of the specimens submerged in seawater solution during the test are shown in Figure 12a–c. These SEM images reveal corrosion pits, intergranular cracks, and induced second-phase particles, indicating that materials corroded. Intergranular corrosion was discovered in base alloy AA8011 when it was submerged in solution. Pitting was discovered to be a problem for base alloy 6061. The weld nugget area was not corroded, indicating that it was likely cathodically shielded by base material 8011, which showed pitting. Corrosion was found to be minimal in the weld region (Figure 12c) when compared to the base material specimens AA6061 (Figure 12a) and AA8011 (Figure 12b). The picture showed little corrosion impact, despite the presence of a localized pit and mild corrosion attack on the weld area specimen. SEM pictures of specimens exposed to an H_2_SO_4_ submersion test are shown in Figure 13a–c. According to SEM images of the specimens, the deep corrosion generated highly localized pit dissolution and created second-phase particles. All of the images show modest corrosion attacks in the weld area (Figure 13c). The AA8011 specimen was severely corroded (Figure 13b), whereas the AA6061 specimen was mildly corroded (Figure 13a). SEM images of the three specimens (AA6061, AA8011, and weld area) that underwent corrosion testing in HCl solution are shown in Figure 14a–c. The damaged surfaces of the specimens displayed corrosion pits, extensive corrosion, and the formation of tiny grain boundaries. Deep corrosion, grooves, and corrosion pits were more significant for the base materials of AA6061 (Figure 14a) and AA8011 (Figure 14b) as compared to the weld region (Figure 14c). In the fine-grained portion of the weld region, AA8011 deteriorated predominantly through pitting and intergranular corrosion. This might have been linked to changes in the microstructures of the TMAZ and HAZ during FSW. The fine-grained portion of the weld region deteriorated predominantly through pitting and intergranular corrosion.

## 4. Conclusions

The corrosion behaviors of two dissimilar alloys (AA6061 and AA8011) were investigated using the FSW technique under a variety of corrosion conditions simulating marine applications. The findings of this investigative process are outlined below.

The weight loss at the weld site was much lower in the salt spraying and submersion tests than for the base materials AA6061 and AA8011. The specimens were submerged in three different media for 28 days, namely 3.5 wt% H_2_SO_4_ solution, 3.5 wt% HCl solution, and seawater. The corrosion was highest in H_2_SO_4_ solution, followed by the HCl solution and seawater. The CR in the weld zone was found to be greater than in the base materials (AA6061and AA8011). According to the SEM investigation, intergranular corrosion, deep corrosion, localized pit dissolution, and corrosion pits were found in more sites of the specimens in acidic solutions than in alkaline solutions (seawater). When compared to the base materials, corrosion testing in acid and alkaline media revealed that the weld region specimen had the lowest corrosion rate. Microstructural alterations in the heat-affected zones resulted in increased corrosion attacks. However, due to differences in the pitting potentials throughout the FSW, galvanic corrosion can occur, especially under harsh alkaline and acidic conditions. As a result, conversion layer coatings are extremely attractive for corrosion protection of these welds.

Corrosion experiments using various FSW settings for the welding of dissimilar connections, as well as various tool profiles and materials, will be possible in the future. This research proposal might be expanded to include a thermal examination of the tool–material interaction during welding using temperature sensors. The FSW principle has recently been modified into friction stir processing (FSP), which has a wide variety of industrial applications for surface modifications. More effort in this field is needed to increase the quality of FSP. The welding rate, axial force, and driving tool rotation speed were all evaluated in this study. The plunge depth, on the other hand, was not assessed, even though this is a crucial aspect when assessing weld quality.

## Figures and Tables

**Table 1:** Chemical patterns showing wt.% and MP values for AA6061 and AA8011 alloys.

Material	Mg	Mn	Cu	Fe	Si	Cr	Zn	Ti	Al	Ultimate Tensile Strength (MPa)	Yield Strength (MPa)	Hardness (HRB)
AA6061	0.84	0.40	0.24	0.70	0.54	0.25	0.20	0.10	Remaining	310	276	60
AA8011	0.27	0.45	0.12	0.74	0.52	0.02	0.08	0.01	Remaining	110	87	50

**Table 2:** FSW parameters.

S. No	FSW Parameter	Value
1.	Rotating speed of tool	1100 rpm
2.	Welding rate	50 mm/min
3.	Axial force	3.5 kN
4.	Tool tilt angle	2°

**Table 3:** Salt spraying test parameters and values.

S. No	Test Parameter	Value
1.	Test solution	5 wt.% NaCl
2.	Chamber temperature	34 °C
3.	pH of salt solution	6.8
4.	Collection of solution per hour	1.1 mL
5.	Air pressure	15 Psi
6.	Exposure period	60 h

**Table 4:** Submersion test media and their details.

No	Solution	pH Value	Temperature (°C)	Submersion Time (Days/hours)
1	3.5 wt% HCl + H_2_O	3.08	32	28/672
2	3.5 wt% H_2_SO_4_ + H_2_O	3.51		
3	Seawater	7.52		

**Table 5:** Summary of the salt spraying test.

Specimen	Solution	Weight of the Specimen before Submersion (g) W_b_	Weight of the Specimen after Submersion (g) W_a_	Weight Loss (g) ΔW	Corrosion Rate (mm/yr) Cr
AA6061	5 wt% NaCl	11.5	11.6474	0.1476	0.00324
AA8011		11.51	11.75	0.24	0.00516
Weld Zone		11.42	11.5312	0.1212	0.00264

**Table 6:** Summary of the submersion test.

Specimen	Solution	Weight of the Specimen before Submersion (g) W_b_	Weight of the Specimen after Submersion (g) W_a_	Weight Loss (g) ΔW	Corrosion Rate (mm/yr) Cr
AA6061	3.5 wt% HCl + H_2_O	11.5	9.772	1.728	0.04326
AA8011	3.5 wt% HCl + H_2_O	11.51	9.554	1.956	0.0488
Weld Zone	3.5 wt% HCl + H_2_O	11.42	9.776	1.644	0.0411
AA6061	3.5 wt% H_2_SO_4_ + H_2_O	11.5	9.86	1.64	0.0411
AA8011	3.5 wt% H_2_SO_4_ + H_2_O	11.51	9.71	1.8	0.0449
Weld Zone	3.5 wt% H_2_SO_4_ + H_2_O	11.42	9.956	1.464	0.0366
AA6061	Seawater	11.5	11.218	0.282	0.00706
AA8011	Seawater	11.51	11.216	0.294	0.00733
Weld Zone	Seawater	11.42	11.216	0.204	0.00510

## Data Availability

The data underlying this article will be shared on reasonable request from the corresponding author.
